# The environment and physical activity: The influence of psychosocial, perceived and built environmental factors

**DOI:** 10.1186/1479-5868-6-19

**Published:** 2009-03-30

**Authors:** Ralph Maddison, Steven Vander Hoorn, Yannan Jiang, Cliona Ni Mhurchu, Daniel Exeter, Enid Dorey, Chris Bullen, Jennifer Utter, David Schaaf, Maria Turley

**Affiliations:** 1 Clinical Trials Research Unit, School of Population Health, Faculty of Medical and Health Sciences, University of Auckland; 2 Epidemiology and Biostatistics, School of Population Health, University of Auckland; 3 Pacific Health, School of Population Health, University of Auckland

## Abstract

This study sought to integrate perceived and built environmental and individual factors into the Theory of Planned Behavior (TPB) model to better understand adolescents' physical activity.

Participants (n = 110) aged 12 to 17 years (M = 14.6 ± 1.55) were recruited from two large metropolitan high schools in Auckland, New Zealand, were included in the analysis. Participants completed measures of the revised TPB and the perceived environment. Individual factors such as ethnicity and level of deprivation were also collected. Geographical Information Systems (GIS) software was used to measure the physical environment (walkability, access to physical activity facilities). Physical activity was assessed using the ActiGraph accelerometer and the Physical Activity Questionnaire for Adolescents (PAQ-A). Data from the various sources were combined to develop an integrated model integrated for statistical analysis using structural equation modeling.

The TPB model variables (intention and perceived behavioral control) explained 43% of the variance of PAQ-A. Unique and individual contributions were made by intention and PBC and home ownership of home equipment. The model explained 13% of time spent in moderate and vigorous physical activity (Actigraph). Unique and individual contribution was made by intention.

Social cognitive variables were better predictors of both subjective and objective physical activity compared to perceived environmental and built environment factors. Implications of these findings are discussed.

## Introduction

### Environmental influences on children's activity

Recent increase in global obesity rates has been proposed to be related to changes in our physical and social environments, which increasingly promote a high energy intake and sedentary behaviors [1]. The importance of supportive environments that promote healthy choices is enshrined within the Ottawa charter [2], and more recently, sophisticated environmental models have been developed that describe the interdependent influences of biological, social, behavioral and environmental factors on diet [3] and physical activity [4], which are key mediators for obesity [5]. Despite these proposed models, few studies have investigated the interrelationship between these factors.

In recent years there has been increased research interest in the potential impact of environmental factors on both nutrition and physical activity. A review of 19 studies [6] demonstrated consistent associations between physical activity and perceptions of accessibility, opportunities, and the aesthetics of the environment among adults. Environmental studies have also reported positive associations between walking and access to open space and high neighborhood walkability [7], whereas increased cycling was associated with absence of busy streets [8] and the presence of green and recreational space [9]. Generally the relations between environmental factors and physical activity have been small in magnitude and often conducted in studies with large sample sizes; and most have focused on physical activity in adults [10-14] and have largely overlooked children and adolescents [15-18]. Environmental factors found to be related to physical activity in children and adolescents include greater access to recreational facilities, greater opportunities to exercise, increased time spent outside [19-21]. High crime rates, personal safety concerns [21,22], and transport infrastructure (number of roads to cross and traffic density/speed) [21] have been found to be related to decreased levels of physical activity.

### Environmental and psychosocial factors: a combined approach?

Ecological models posit that behaviors have multiple levels of influence that include intrapersonal, interpersonal, policy, and environmental variables [23]. Therefore, a combination of psychosocial and environmental variables should best explain physical activity behavior [4,24,25]. Environmental research studies, if they are to be of practical use in public health policy, ought to focus on the environmental influences that may determine particular behavioral choices [17,18,26]. Understanding the interaction of an individual with the environment is just as important as measuring the environment itself. Therefore combined research of psychosocial and environmental influences on these health-related behaviors is necessary to target interventions appropriately. However, few studies to date have examined both psychosocial (perceptions) and environmental factors in relation to physical activity. One cross sectional study of 235 Australian children aged 5–6 years and 677 children aged 10–12 years [18] found that both environmental and social factors were related to physical activity (active commuting to school).

Recent research studies have examined the relative contribution of psychological, social, individual, and environmental factors on physical activity behaviors and have shown that after adjustment for other determinants, exercising according to recommendations was more strongly associated with individual determinants than other social environmental or physical environmental determinants [27]. Others have shown that perceived access to opportunities for physical activity, and motivation to be active were associated with regular vigorous activity among adolescents [28]. Despite these findings, the evidence regarding environmental determinants of energy balance related behaviors (e.g., physical activity and nutrition) has been largely based on non-theoretical approaches [29]. Recently, researchers have examined the influence of environmental variables on walking behavior using the Theory of Planned Behavior (TPB) [30] model as a framework. According to the TPB, intention is proposed to be the most immediate determinant of behavior. The constructs attitude, subjective norm, and perceived behavioral control (PBC) are proposed to influence intention. PBC is also posited to directly influence behavior. External factors such as the environment are considered antecedents of the social cognitive factors and should be mediated by these constructs. External factors may also moderate the relationship between the social cognitive factors and behavior. In two separate studies with Canadian adults, the effect of environmental factors (e.g., neighborhood aesthetics, retail land-use mix) on walking behavior was mediated by attitudes [31,32] and intention [32]. Perceived proximity to recreation facilities also moderated intention-walking relations, with those perceiving a closer proximity showing a larger intention-walking relationship than those who reported being farther away from recreation infrastructure [31].

To the best of our knowledge only one study has modeled individual and perceived environmental factors in addition to the TBP among adolescents [33]. Results showed that past physical activity had the strongest relationship with current self-reported physical activity, while perceived environmental aesthetics and distance to activity opportunities were indirectly related to adolescents' intentions to be physically active. The model explained 17% of the response variance of physical activity.

No studies have yet integrated perceived and measured environmental factors with psychological constructs from the TPB to determine their contribution to physical activity behavior among adolescents. The aim therefore of this study was to examine the feasibility of integrating environmental, individual, and psychological variables to better understand physical activity in New Zealand (NZ) adolescents. Specifically, this study sought to integrate perceived and built environment measures with the TPB model. Based on previous research [32] we hypothesized that environmental factors would be correlates of physical activity but mediated through attitudes and PBC and subsequent intentions to be active.

## Methods

### Participants and design

Participants were students at two large Metropolitan schools in the Auckland region of NZ, aged between 12 and 17 years. All study procedures and related documents were approved by the regional ethics committee. Contact was initiated with the school principal to discuss the study purpose and procedures and to obtain consent to approach the students. Once permission was obtained, a researcher made contact with the students via a designated teacher. Students were provided with written and verbal information about all aspects of the study and offered the opportunity to participate. Interested participants completed informed consent forms. Parental consent was also obtained for adolescents aged < 16 years. Data collection took place during designated classroom time. Participants completed measures of the TPB model (attitude, subjective norm, PBC, and intention), perceived environment, and self-reported physical activity. At the end of the session, students were fitted with an Actigraph accelerometer, which they were instructed to wear on their right hip, during all waking hours over 4-consecutive days (including 2 weekend days). Exceptions to this rule were (a) all water sports and showering (as the Actigraph is not water resistant) and (b) any contact sports (e.g., Rugby Union). Height and weight also were measured. Researchers returned to the school after four days had elapsed to collect the accelerometers.

In total, 170 school students registered interest to participate, of which 164 were eligible and took part in the study. Six were ineligible because of age. In NZ, schools have boundaries or zones, in which students must live to attend the school. It is possible to attend the school if you live outside the zone under certain circumstances (siblings attending the school etc.). One hundred and ten students resided within the respective school zones for which Geographic Information Systems (GIS) data were available and were included in analysis. On average, these students were 14.6 years of age (SD 1.55), overweight (mean BMI 24.7 kg/m^2^, SD = 6.7), predominantly male (57%) and mixed ethnicity. Demographic details are provided in Table 1 for the total sample and for each school.

**Table 1:** Descriptive information of all participants

	Variable	*M*	*SD*
Age (years)		14.61	1.55
Height (meters)		1.66	0.1
Weight (kg)		69.06	22.73
BMI		24.7	6.65
NZDep		6.33	3.14

		*n *(%)	

Sex	Male	64 (58%)	
	Female	46 (42%)	

Ethnicity	NZ European	39 (35.5%)	
	NZ Maori	16 (14.5%)	
	Pacific	29 (27.3%)	
	Asian	14 (12.7%)	
	Other	12 (10.9%)	

N = 110			

### Measures

#### Body composition

Height (Harpenden Stadiometer, Chasmors Ltd, London) weight (Salter scales), and waist circumference were measured according to standardized procedures [34].

#### Revised TPB variables

All TPB items were constructed according to Ajzen's recommendations [35] and to ensure suitability for an adolescent sample and have been used in previous research with this age group [36]. Researchers administered all questionnaires. For the TPB questions, regular physical activity was defined as going any physical activities for a total of 60-minutes or more per day (on most days of the week). This definition is based upon New Zealand's physical activity guidelines for children and young people.

*Intention *was assessed using three items, "I intend to take part in regular physical activity next week"; I plan to do regular physical activity next week"; and I will try to take part in regular physical activity next week. Items were rated using a 7-point Likert scale, from -3 (completely disagree) to +3 (completely agree). An overall measure of intention was generated from the mean of the three items, with higher scores indicating greater intent to be physically active.

*Attitude *towards physical activity was assessed using the stem item "For me to take part in regular physical activities during the next week is ...". Six bipolar adjective scales were scored from 1 to 5. The scales included both experiential (e.g. *enjoyable-not enjoyable, pleasant-unpleasant, fun-boring*) and instrumental (e.g. *useful-useless, harmful-beneficial*) items. The instructions that preceded the adjectives directed the participant to "*Please put a tick on the line for the amount that you agree or disagree with the statement*". To obtain an overall measure of a participant's attitude each of the five items were summed. Possible scores for the overall attitude measure could range from 5 to 30.

*Subjective norm *was assessed using three items. One item assessed descriptive norms and two items assessed injunctive norms. Descriptive norms were assessed with the item "People who are important to me (e.g., mum and dad, guardian, grandparents, other family, friends, teachers etc) want me to participate in regular physical activities over the next week'. Injunctive norms were assessed with the items, "Most of my friends take part in regular physical activities"; and "My mum/dad (guardian) take part in regular physical activities". Items were rated on a 7-point scale, from -3 (completely disagree) to +3 (completely agree). An overall measure of subjective norm was generated from the mean of the three items.

*Perceived behavioral control (PBC) *was measured using three items "For me to take part in regular physical activities in the next week would be" -3 very difficult to +3 very easy; "Whether I do or do not take part in regular physical activity next week is completely up to me" (-3 completely false to +3 completely true); "How much control do you have in taking part in regular physical activity next week", rated from -3 (no control) to +3 (complete control). A mean score from the three items was calculated to give an overall measure of PBC. Internal consistency of all scales was high (range a = 0.75 – 0.97).

#### Perceived environment

Items from the Neighborhood Environments Walkability Scale (NEWS) [37-39] were used to assess perceptions of access to physical activity facilities as well as ownership of household-items for recreational use. There is currently a lack of standardized measurement of the perceived environment, however these measures highlight the proximity of facilities, crime, aesthetics, and safety as key characteristics to walking and cycling [7,18,32]. Given the importance of perceived access to facilities and physical activity resources we chose to use the relevant NEWS items. Some of the wording was modified to ensure applicability to the NZ setting (e.g., sidewalks was changed to footpaths, local shop was changed to dairy).

#### Perceived access

Participants indicated the duration (in minutes) it would take them to walk from their home to the closet of 17 facilities for the purpose of physical activity (e.g., public park, recreation centre, playground etc.). Walking time to facilities was categorized as 1–5 minutes, 6–10 minutes, 11–29, 21–30, and 30+ minutes. The categorical variable was used in analysis.

#### Perceived ownership and reported use of equipment

Participants indicated (by ticking a yes or no option) the type of equipment they had in their home or garden for the purpose of physical activity (e.g., bicycle, swimming pool, skates etc). Participants also reported the number of times they had used each item of equipment for the purpose of physical activity in the previous month. The sum of the number of items owned, and the number of items used, respectively, were used for analysis.

#### Perceptions of safety

Specific items were developed to assess participants' perceptions of safety and difficulty for walking and cycling. Items were based on previous work with Australian children by Timperio and colleagues [18]. Participants indicated the level to which they disagreed with nine statements regarding the difficulty to walk and cycle in their neighborhood (e.g., there is a high crime rate in my neighborhood). Items were scored on a scale from 1 (strongly disagree) to 5 (strongly agree).

*Physical activity behavior *was assessed subjectively by self-report and objectively by accelerometer. Research has shown that it is particularly challenging to obtain reliable and valid measurements of youth PA [40]. Collecting PA data objectively and subjectively allows for comparison between the two methods.

Participants completed the Physical Activity Questionnaire for Adolescents (PAQ-A), a validated self-report seven-day recall physical activity measure, consisting of nine items that are used to calculate summary activity scores. Items assess physical activity performed at school (physical education, recess, lunchtime), right after school, and at home (organized and recreational) [41]. Each PAQ-A statement was scored on a five-point scale with higher scores indicating higher activity levels.

Previous research [42] has reported mean scores of 3.23 (SD 0.78) and 3.35 (0.68) in young Canadians. Participants were also fitted with an Actigraph Accelerometer (Model AM7164-2.2C), which measures motion in the vertical plane, with movement outside of 'normal' motion being filtered electronically. Students wore the accelerometer during waking hours on their right hip at the mid-axilla line and activity count data (counts/minute) were collected over four consecutive days (two weekdays and two weekend days). Minute-by-minute activity counts were uploaded to a data reduction program that excluded all Actigraph outputs that equaled zero for more than 20 consecutive minutes (assuming non-wearing time for that period). All days with less than ten hours of recorded time were excluded from analyses. Using these criteria, all participants provided at least three valid days (including one weekend day) for analysis. Time spent in light (≤ 2.9 METS), moderate (3.0–5.9 METS), and vigorous (≥ 6.0 METS) activities were derived from age-specific count cut-offs developed by Freedson et al [43].

#### Built environment

Geographical Information Systems (GIS) (ARCGIS) [44] was used to map neighborhood environments (for those living within the school zoning area) in terms of the number, type, and location of activity opportunities (recreation facilities, parks etc) as well as to determine walkability. The addresses of registered recreational facilities (gyms, sports clubs, public swimming pools etc.) were derived from secondary data sources (Auckland City Council, Auckland Public Health Service, Auckland Regional Council, and the APNfinda) and imported to a GIS database. Green Space and land use data were derived from the Auckland City Council district plan. The GIS database also included New Zealand census and deprivation index data. From these GIS data we generated two environmental indices.

#### Accessibility Index

The construction of the indices was based on previous accessibility measures [45]. Within each neighborhood, accessibility to each type of facility or location (e.g., playground, swimming pool etc.) was calculated by counting the number of facilities within a specified time threshold from the participant's house. We used the roads network and the assumption that walking speed was 4 km/hr, and thresholds were defined as <5 minutes, 6–10 minutes, 11–29, 21–30, and 30+ minutes, to be comparable with the results from the participant's perceived accessibility. The GIS data were integrated with the key facilities identified in the 'perceptions of the environment' questionnaire to derive the distance of decay parameters, which was measured as:



Where *A*_*i *_is a measure of accessibility from each unique mesh-block i, mj is set to 1 for facilities (in the absence of data for 'attractiveness' of each facility) and b is the estimated destination-specific distance-decay parameter between i and j.

#### Walkability index

A walkability index was developed to indicate the ease of walking around a neighborhood and was based on dwelling density, street connectivity, and land attributes. Because the index was originally developed in the United States of America modifications were made to ensure specificity for NZ. A 1–10 score for each measure (dwelling density, intersection density, and land use) was summed for each meshblock resulting in a possible score of 3–30.

#### Deprivation index (NZDep)

The NZDep2001 index of combines nine variables from the 2001 census which reflect eight dimensions of deprivation. NZDep2001 provides a deprivation score for each meshblock in NZ. Meshblocks are geographical units defined by Statistics NZ, containing a median of approximately 90 people in 2001. The NZDep2001 scale of deprivation from 1 to 10 divides New Zealand into tenths of the distribution of the first principal component scores. For example, a value of 10 indicates that the meshblock is in the least deprived 10 percent of areas in NZ.

### Analyses

Structural equation modeling was carried to determine associations between the environmental variables (GIS and perceived environment), TBP variables and physical activity. Structural equation modeling analyses were performed using AMOS 4.0 [46]. All other analyses were conducted using *SAS *version 9.1.3 (SAS Institute Inc. Cary NC). All statistical tests were two-tailed and a 5% significance level maintained throughout the analyses.

In line with previous research [32] to create a more parsimonious and less complex model the following approaches were taken. Bivariate correlations of perceived environment, individual, and built environment variables with measures of physical activity (PAQ-A, and time spent in MVPA) were evaluated. Only significant (p < 0.05) bivariate correlations between these variables were integrated to predict each measure of physical activity. For the TPB variables, established theory was used to identify proposed relationships with physical activity.

Structural equation modeling [47] with maximum likelihood estimation and a covariance matrix was used to test the integrated model. Given the exploratory nature of this research and the complexity of the model we proposed five separate levels. Level I was the dependant variable (physical activity). Consistent with the TPB, intention was proposed as the most proximal determinant of physical activity (level II) with PBC, PSN, and attitude antecedents of intention (level III). According to the Theory of Planned Behavior external factors are posited to be antecedents of the social-cognitive variables, however, it is possible these variables may also have a direct relationship to physical activity. Therefore, in the proposed model perceived environment variables (perceptions of safety, ownership and use of recreational equipment) (Level IV) and the built environment (GIS) variables (walkability and accessibility) (Level V) were proposed to both influence social cognitive factors and behavior directly. Ethnicity and deprivation were also considered at level V, and were set to correlate with each other in the model. However the perceived environment and social cognitive variables were freed to correlate amongst each other. For the latent variables, the loading of the first indicator was set to 1.0. Two models were tested with physical activity measured using the PAQ-A and accelerometer.

## Results

On average, adolescents participated in 74 (SD = 35.96) minutes of moderate and 8 (SD = 8.35) minutes of vigorous physical activity per day. The mean PAQ-A score was 2.8 (SD = 0.81). Descriptive information and bivariate correlations of the variables of interest are presented in Table 1. The social cognitive variables were correlated with the subjective (PAQ-A) and objective physical activity (time spent in moderate/vigorous activity). Perceived environment variables (perceived ownership of recreational equipment and perceived access) correlated with the PAQ-A only. None of the perceived variables were correlated with time spent in MVPA. The GIS variables (walkability and accessibility) were not correlated with any of the physical activity measures. Time spent in MVPA correlated with the PAQ-A (r = 0.50, p < 0.0001).

**Figure 1:**
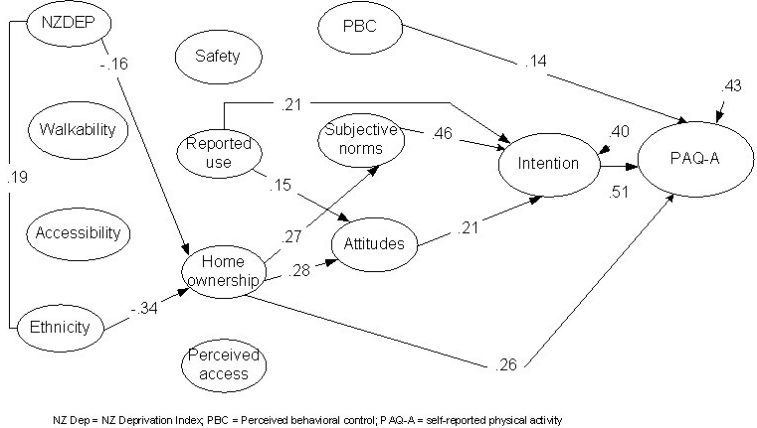
**Integrated model to predict self-reported behavior (PAQ-A)**. Note: All effects are standardized. Bold lines indicated statistically significant relationships (p < 0.05).

Structural equation modeling analysis with the PAQ-A as the dependant variable is presented in Model one (Figure 1). The model resulted in a modest fit of the data [Χ^2 ^(28) = 82.98; p < 0.0001; CFI = 0.79; RMSEA = .13] using conventional cut-off criteria. Approximately 43% variance of subjective physical activity (PAQ-A) was explained. Intention (standardized effect = .51) and PBC (standardized effect = 0.14) had the strongest direct effects on subjective physical activity. Of the perceived variables, home ownership of recreation equipment (standardized effect = .26) had a direct effect on physical activity. Subjective norms and attitude were related to intention, while PBC was not. As can be seen in Figure 1, perceived ownership of home equipment had a direct relationship with all of the TPB variables except PBC, while reported use of home equipment had a direct effect with attitude. Ethnicity (standardized effect = -.34) was inversely related to perceived home ownership of recreational equipment.

**Figure 2:**
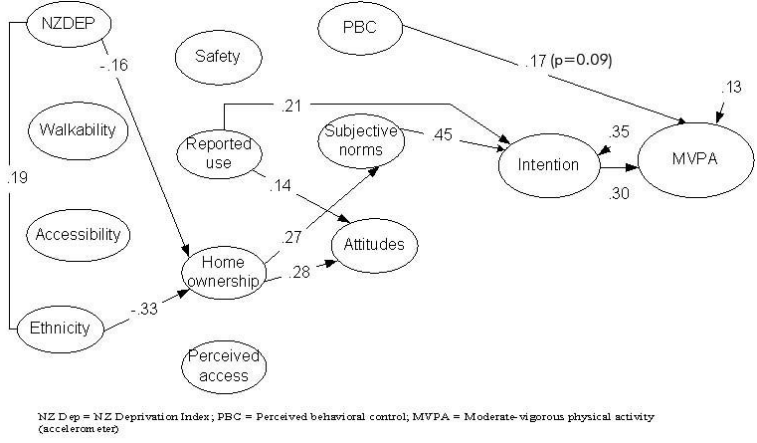
**Integrated model to predict moderate-vigorous physical activity (MVPA)**. Note: All effects are standardized. Bold lines indicated statistically significant relationships (p < 0.05).

A second model (Figure 2) was tested with objective physical activity (accelerometer) as the dependant variable (time spent in MVPA). The model again resulted in a modest fit of the data [Χ^2 ^(29) = 78.04; p < 0.0001; CFI = .70; RMSEA = .12], with approximately 13% of the response variance of objective physical activity explained. Intention (standardized effect = .30) and PBC (standardized effect = .17; p = 0.09) had the strongest direct effects on objective PA. None of the perceived or built environment variables were directly related to accelerometer measured PA.

An alternative model was tested in which the individual variables (ethnicity and NZDep) were freed to correlate; however this did not improve the overall fit (ΔΧ^2 ^(6) = 0.85; p > 0.05).

### Mediation analysis

None of the environmental variables met the criteria for mediation [48].

## Discussion

This study sought to examine the feasibility of integrating environmental (perceived and GIS measured), individual, and psychological variables to better understand physical activity in NZ children. Overall, our results showed that is possible to integrate these variables to examine their contribution to physical activity. In general, results showed that the TPB variables were the most proximal determinants of both subjective and objectively measured physical activity. The perceived environmental variables (reported home ownership and use of sport and recreation equipment) were related to the TPB variables and self-reported PA. Built environment variables were not directly or indirectly related to the PA measures.

Differences were found between the various measures of physical activity. The integrated model explained 43% and 13% of self-reported physical activity (PAQ-A), time spent in MVPA, respectively. The TPB variables contributed most to the prediction of these behaviors. Intention and PBC predicted self-reported behavior (PAQ-A), whereas only intention predicted accelerometer-derived behavior. These findings are consistent with social cognitive theory, which posits that intention is the most proximal determinant of physical activity and that PBC is directly related to behavior as well as intention. The difference in findings between self-reported and objective physical activity suggest that different psychological variables may be salient for different aspects of physical activity. Specifically, it is likely that greater congruence exists between self-report measures of physical activity such as the PAQ-A and the items used to measure constructs such as intention, attitudes etc. Whereas accelerometers capture all activities (including incidental), which may be less volitional and therefore TBP items may be less likely to capture.

Although the two activity measures were correlated (*r *= 0.50, p < 0.01) indicating shared variance, they also clearly assess separate aspects of physical activity. Much of the physical activity assessed with the PAQ-A could be considered structured either by time (i.e. during PE class, straight after school, in the evening, and in the weekend) or by specific activity (i.e. physical education, sports teams, dance etc.). Hence, participants are more likely to remember and report such activities, and forget the spontaneous, unstructured activities (captured by accelerometers).

Only the perceived environmental variables were directly related to the self-report measures of physical activity. Specifically, reported home ownership was directly related to the PAQ-A measure. These finding suggest that greater access to sport and recreation equipment within the home may increase opportunities to be active, which in turn encourages participation in physical activities; or those who intend to be active may have more home equipment. These findings are contrary to those of a systematic review [49], which reported that four out of six studies found no association between home equipment and children's physical activity. In contrast, two studies [50,51] found that the number pieces of exercise equipment in the home was positively and significantly associated with higher self-reported physical activity among adolescents girls and boys and young adolescent girls (but not boys). However availability of sporting equipment (ball, bats, etc,) has been shown to be related to children's physical activity within the school environment [21]. Home ownership was not related to accelerometer measured physical activity. The reason for this is not clear, and may be related to the fact that the systematic bias associated with self-reported ownership is the same associated with self-reported behavior. Therefore congruence in measurement is more likely with self-report than objectively assessed behavior. However, intuitively, it makes sense that access to activity promoting toys is likely to be related to increased physical activity participation. Interestingly, all TPB variables (except PBC) were related to home ownership of sport and recreation equipment.

Perceived safety was not related to physical activity or any of the TPB variables. The lack of association with physical activity is consistent with previous research in children (see Davison [21] for a review). However, among adolescents perceived neighborhood safety was associated with higher self-reported outdoor physical activity for girls but not boys [21] as well as vigorous activity [53]. The lack of support for perceived safety may be reflected in the measurement of general levels of physical activity, which may or may not be linked with neighborhood safety given that children can be active outside their neighborhood. Of more surprise was the lack of relations between perceived safety and the TPB variables. According to theory, we hypothesized that perceived variables would act as antecedents of the TPB variables, which was not the case. A small (r = .12, p = 0.09) correlation was found between perceived safety and PBC, however due to the small sample size this was statistically non-significant.

Contrary to previous research, walkability was not related to self-reported or objectively measured physical activity behavior. There a number of possible explanations for this lack of relationship. First and foremost the walkability of a neighborhood may have very little effect on free-living physical activity behavior. Previous studies in adults have found small correlations between walkability and self-reported walking [7], which may just as likely be a function of large n size. It is also likely that neighborhood walkability may be more related to walking behavior than general levels of physical activity measured (e.g., PAQ-A and accelerometer). Other explanations include the small sample size, and clustering effect of children from similar school classes, which may have diminished the impact of built environmental variables given the close proximity and likely geographical similarity of the neighborhoods

In the present study, the measured accessibility index was not related to any of the physical activity measures. This was surprising given that the index included all neighborhood facilities. The lack of associations between the measured environment factors and physical activity suggest that these variables are not related and physical activity does not necessarily occur in the neighborhoods that people reside. These findings also highlight the lack of sophistication and precision associated with the way we currently measure these environmental variables.

The integrated model included the individual (or demographic) variables ethnicity and deprivation index, which were both inversely related to home ownership of sport and recreation equipment. According to how these variables were coded, Maori and Pacific and those with greater deprivation were less likely to report ownership of sport and recreation equipment in the home, highlighting the issue of disparity of access to opportunities to be active.

According to conventional criteria the model fit was modest at best; however this study sought to assess the feasibility of integrating these data and was limited by the small sample size given the complexity of the proposed model. However, structural equation modeling provides a suitable approach to examine the complex interdependent relations between the social, behavioral, and environmental factors on physical activity. Consistent with theory, our model considered the TPB variables as the proximal correlates of behavior, with external factors such as the perceived and built environment as antecedents of the TPB variables but also as more distal correlates of physical activity. Alternative models could equally have been proposed and tested. For example, Bandura proposed an interrelationship between the person, environment, and behavior (triadic determinism).

This study is not without limitations and should be considered within the context of these. First, this was a feasibility study and therefore had a small sample size given the number of variables examined. Second, participants were from two metropolitan schools in NZ which limits the generalizability of the findings. Third, we used a cross sectional design and therefore cannot infer causation. Notwithstanding these issues, this study used a theoretical approach to integrate perceived and GIS-measured environment, and individual factors within the TPB model to predict physical activity in adolescents.

## Conclusion

A model that integrated the TPB, perceived and GIS-measured environment and individual factors found that self-reported physical activity and MVPA are largely intention and control-based behaviors, with an additional independent contribution from home ownership of sport and recreation equipment. Taken together, these findings suggest that theory-based interventions focused on enhancing intentions, perceived control as well as perceptions of the environment may have positive impacts on physical activity. Future work should focus on developing alternative models where the role of the environmental variables can play a greater role in predicting physical activity.

## Competing interests

The authors declare that they have no competing interests.

## Authors' contributions

RM conceived of the study, and participated in its design and coordination. He was involved in the analysis of data, interpretation of results, wrote the manuscript and prepared subsequent revisions. SV and YJ were involved in developing and refining the study design, data analysis, and interpretation of results. CNM helped develop the study idea, design and methods. She was involved in the interpretation of results. DE conducted the GIS analysis and was involved in the interpretation of results. ED was involved in the study coordination and data collection. CB, JU, DS, MT were all involved in refining the study design and methods. All authors read and approved the final manuscript.

## Note

Table 3

Correlations of social cognition, perceived environment, built environment and individual factors

**Table 2:** Correlations of social cognition, perceived environment, built environment and individual factors with physical activity

	Mean	SD	PAQ-A	MVPA
Intention	1.70	1.30	.66**	.38**
Perceived behavioral control	1.64	1.12	.36**	.28**
Attitudes	4.30	0.71	.45**	.31**
Subjective norm	1.31	1.23	.48**	.35**
Perceived safety	3.32	0.66	-.01	-.01
Reported ownership of home equipment	4.75	2.41	.42**	.12
Reported use of home equipment	0.73	0.29	.23*	.06
Perceived access	15.73	4.53	-.26**	-.01
Walkability	6.14	3.28	-.04	-.03
Accessibility	1.18	0.45	.02	-.08
NZ Deprivation (NZDEP)	6.33	3.14	.06	-.01
